# Is Burnout Primarily Linked to Work-Situated Factors? A Relative Weight Analytic Study

**DOI:** 10.3389/fpsyg.2020.623912

**Published:** 2021-01-13

**Authors:** Renzo Bianchi, Guadalupe Manzano-García, Jean-Pierre Rolland

**Affiliations:** ^1^Institute of Work and Organizational Psychology, University of Neuchâtel, Neuchâtel, Switzerland; ^2^Departamento de Ciencias de la Educación, Universidad de La Rioja, Logroño, Spain; ^3^Unité de Formation et de Recherche des Sciences et Techniques des Activités Physiques et Sportives, Université Paris Nanterre, Nanterre, France

**Keywords:** burnout, depression, job stress, neuroticism, personality, relative weight analysis

## Abstract

It has often been asserted that burnout is primarily linked to occupational-context factors, and only secondarily to individual-level (e.g., personality) and non-work (or general) factors. We evaluated the validity of this view by examining the links between burnout and an array of 22 work-situated (effort-reward imbalance, unreasonable work tasks, unnecessary work tasks, weekly working hours, job autonomy, skill development, performance feedback, and support in work life), work-unrelated (sentimental accomplishment, familial accomplishment, number of child[ren], leisure activities, residential satisfaction, environmental quality, security in daily life, and support in personal life), dispositional (neuroticism, sex, age, and physical condition), and intersecting (work–non-work conflict and non-work–work conflict) variables. The study involved schoolteachers from three different countries: France (*N* = 4,395), Spain (*N* = 611), and Switzerland (*N* = 514). Burnout was assessed with the Maslach Burnout Inventory for Educators. Most of our predictors were assessed based on widely used measures (e.g., neuroticism was assessed with the NEO-Five Factor Inventory). In order to assess sentimental accomplishment and familial accomplishment, we created two self-reported measures, namely, the Sentimental Accomplishment Inventory (SAI; 9 items) and the Familial Accomplishment Inventory (FAI; 9 items). The SAI and the FAI both showed strong reliability and high factorial validity. Exploratory structural equation modeling bifactor analysis and Mokken scaling suggested that both instruments could be considered essentially unidimensional. The study results showed that neuroticism, job strain, skill development, security in daily life, and work–non-work conflict were consistently associated with burnout across the three samples. Sample-specific predictors of burnout included sex, age, unreasonable work tasks, weekly working hours, job autonomy, support in work life, sentimental accomplishment, leisure activities, support in personal life, and non-work–work conflict. Relative weight analysis indicated that neuroticism was the best predictor of burnout in each sample. Our findings suggest that burnout’s nomological network may not be primarily job-related. We conclude that the tendency to de-emphasize individual-level and non-work factors in burnout research is unwise. This tendency may constitute a roadblock in the development of effective interventional strategies. The implications of our findings for burnout’s conceptual status are discussed. The neuroticism-burnout link should be further examined in longitudinal studies.

## Introduction

Burnout has been generally regarded as an occupational syndrome combining emotional exhaustion, depersonalization, and a sense of diminished personal accomplishment (Schaufeli and Enzmann, 1998; Maslach et al., 2001). Emotional exhaustion refers to the feeling of being stressed out and drained of one’s energy at work. Depersonalization reflects a state of resentful detachment vis-à-vis one’s activity and a tendency to objectify the persons connected with one’s work (e.g., students, patients, clients). Diminished personal accomplishment involves a sense of ineffectiveness and failure in the job. From an etiological standpoint, burnout has been considered a product of insurmountable job stress (Maslach et al., 2001). There is evidence, for instance, that increases in job demands (e.g., workload) and decreases in job resources (e.g., job autonomy), which can give rise to unmanageable difficulties at work, predict burnout (Schaufeli et al., 2009; Hakanen et al., 2019). A systematic review and meta-analysis of 25 prospective/case-control studies conducted by Aronsson et al. (2017) concluded that job support and workplace justice were protective against burnout symptoms whereas high job demands, low job control, high workload, low job reward, and job insecurity fostered the development of burnout symptoms. Interestingly, an even more recent meta-analysis revealed that burnout increases job stressors much more than vice versa (Guthier et al., 2020).

Although (a) insurmountable stress is best understood through the interplay between the individual and his or her environment (Goldstein and Kopin, 2007; Chrousos, 2009; Pryce et al., 2011) and (b) burnout has been suggested to involve a misfit between the person and the job (Maslach et al., 2001), it has been persistently claimed that the development of burnout was preponderantly driven by occupational-context factors, with individual-level and non-work (or general) factors regarded as non-critical to the emergence of the syndrome. Thus, Brock-Utne and Jaffe (2020) suggested that “burnout is largely a result of the policies of institutions, not a lack of stress management skills or a failure to maintain a proper work-life balance” (p. 334). According to Shanafelt et al. (2017), burnout “is primarily a system-level problem driven by excess job demands and inadequate resources and support, … not an individual problem” (p. 1828). In Dyrbye and Shanafelt’s (2016) opinion, “[f]actors within the learning and work environment, rather than individual attributes, are the major drivers of burnout” (p. 132). In a similar vein, Maslach and Leiter (2010) affirmed that “job variables and the organizational context are the prime predictors of burnout” (p. 728). Maslach (2003) advanced the view that “burnout is more a function of the situation than of the person” (p. 191). Maslach et al. (2001) alleged that the relationships between individual characteristics and burnout “are not as great in size as those for burnout and situational factors” (p. 409). Such claims have been widely endorsed in burnout research. Their validity, however, has been called into question on both theoretical and empirical grounds (e.g., Swider and Zimmerman, 2010; Bianchi and Schonfeld, 2016; Bianchi, 2018; Prins et al., 2019).

The tendency to de-emphasize the role of individual-level and non-work factors in burnout research is intriguing for at least three reasons. First, the importance of individual differences in burnout is rendered salient by the basic fact that, subjected to similar working conditions and obligations within the same workplace, some workers develop burnout symptoms whereas others do not (Bianchi et al., 2018a). In keeping with this observation, meta-analytic studies have revealed strong true score correlations between burnout and the Big Five personality traits, most notably *neuroticism* (Alarcon et al., 2009; Swider and Zimmerman, 2010; You et al., 2015). Neuroticism is a broad and basic dimension of personality that captures the propensity to experience negative, distressing emotions (e.g., sad mood, anxiety, anger/hostility, and helplessness) and to exhibit the associated cognitions and behaviors (e.g., low self-esteem, inhibition of action, threat-related bias; Costa and McCrae, 1987; Brandes and Tackett, 2019; Berggren and Eimer, 2020). Neuroticism has been linked to psychopathology in general and depression and suicide in particular (Kotov et al., 2010; Hakulinen et al., 2015; Jeronimus et al., 2016; Batty et al., 2018). In a recent study, neuroticism was found to account for more variance in burnout than job-related effort-reward imbalance and social support at work combined (Bianchi, 2018). On a related note, neuroticism has been causally linked to job dissatisfaction (Rukh et al., 2020).

Second, dispositional characteristics are widely recognized to influence the way individuals construct their (occupational) reality, at both perceptual and conceptual levels (e.g., Buchanan and Seligman, 1995; Bandura, 1999, 2000; Spector et al., 2002; Proffitt, 2006). Thus, for instance, a given (work-situated) stimulus can be appraised as a stressor by some individuals and not by others (Scherer et al., 2001). Unsurprisingly, there is ample evidence that personality contributes to shaping the experience of one’s work environment in general and the perception of job strain in particular (e.g., Törnroos et al., 2013). Personality substantially predicts “subjective” (e.g., job satisfaction) *and* “objective” (e.g., educational attainment, occupational status, and income) work-related outcomes (Magnus et al., 1993; Roberts et al., 2003; Bleidorn et al., 2019).

Third, because difficulties encountered in non-work domains of life can affect individuals’ (coping) resources at work, it is reasonable to assume that non-occupational factors can foster the development of burnout symptoms. In support of this view, off-job activities as well as a number of other non-work factors (e.g., factors related to sentimental and familial life) have been associated with work-related fatigue and engagement (Bekker et al., 2005; ten Brummelhuis and Bakker, 2012; Mather et al., 2014; Marchand et al., 2015; Verweij et al., 2017; Garrick et al., 2018; Müller et al., 2018; McKee-Lopez et al., 2019; Klusmann et al., 2020). Theoretically speaking, virtually any source of stress-either work-situated or not-can be expected to contribute to burnout (Bianchi et al., 2014).

To date, the state of the art thus suggests that the tendency to downplay the significance of individual-level and non-work/general factors in burnout research might be problematic. However, more research has been called for in order to clarify the kind of ill-being and suffering that is captured by the burnout construct (Hakanen and Bakker, 2017). Indeed, studies that examined the nomological network of burnout using multi-domain approaches remain relatively rare and have been marked by non-negligible limitations. First, studies that examined a relatively large range of burnout predictors did not employ analytic techniques allowing for a clear hierarchization of the predictors’ importance (e.g., Marchand et al., 2015). Second, studies that relied on such techniques (e.g., relative weight analysis) focused only on a relatively small number of predicting variables, notably a relatively small number of work-situated factors (e.g., Bianchi et al., 2018a). Third, little attention has been paid to the “triviality trap” in past research (Kasl, 1978). In self-report studies, the triviality trap manifests itself when the items of the measures of the independent and dependent variables show content overlap. A consequence of the triviality trap is the production of inflated, when not entirely spurious, correlations between the predicting and predicted variables. As noted by Schaufeli and Enzmann (1998) and a number of other authors (e.g., Guglielmi and Tatrow, 1998; Bianchi et al., 2018c), the triviality trap has been a significant problem in burnout research. To give but one example, while psychological job demands have been found to predict burnout, psychological job demands are often assessed with items, such as the “work hard” and “excessive work” items of the Job Content Questionnaire (Karasek, 1985), that explicitly overlap with burnout scale items such as the “I feel I’m working too hard on my job” item of the Maslach Burnout Inventory (MBI; Maslach et al., 2016).

In this study, we reexamined the view that occupational-context factors outweigh individual-level and non-work factors in predicting burnout. We framed our study within the *multilevel determinants of workers’ mental health model*, which “integrates work, non-work and individual factors” to the investigation of workers’ mental health (Marchand et al., 2015, p. 445). As per the multilevel determinants of workers’ mental health model, the determinants of worker’s mental health are to be scrutinized through multiples lenses, from the most microscale (e.g., individual or infra-individual level) to the most macroscale (e.g., society as a whole) levels; meso-level structures include entities such as the family, the social network, and the workplace. Consistent with this framework, we investigated the links between burnout and an array of 22 work-situated, work-unrelated, dispositional, and intersecting (e.g., work–non-work conflict; Amstad et al., 2011) factors. We did so in three different samples from three different countries. We selected our factors of interest based on empirically established and/or theoretically likely associations with burnout. We focused on neuroticism, sex, age, physical condition, job strain, unreasonable work tasks, unnecessary work tasks, weekly working hours, job autonomy, skill development, performance feedback, support in work life, sentimental accomplishment, familial accomplishment, number of child(ren), leisure activities, residential satisfaction, environmental quality, security in daily life, support in personal life, and both work–non-work conflict and non-work–work conflict. We relied on relative weight analysis to hierarchize the importance of the predictors of interest (Tonidandel and LeBreton, 2015). Attention was paid to the triviality trap in the selection process of our measures. We made efforts to operationalize our independent and dependent variables based on measures showing no explicit item overlap. A better understanding of the nomological network of burnout is important for characterizing the syndrome more accurately and deal with it more effectively.

## Materials and Methods

### Study Sample and Recruitment Procedure

Our study involved schoolteachers employed in France, Spain, and Switzerland. Schoolteachers have constituted an object of focal concern in burnout research (Maslach et al., 2016; Schonfeld et al., 2017), consistent with the observation that educational staff members are exposed to chronic forms of job stress (e.g., Schonfeld, 2001; Friedman, 2002; Longobardi et al., 2019; see also Gallup Inc., 2014). To recruit our participants, we sent emails containing a description of the study and a weblink to our survey to educational departments and school administrators in the three targeted countries. The contacted departments and administrators were asked to circulate our email to the teachers working in their establishments. Participation in the study was voluntary. Full confidentiality was guaranteed to participants. When required, we requested local authorizations for conducting the study. Schoolteachers were invited to take part in the study whether experiencing job stress or not. In France, 4,395 schoolteachers responded to the survey; in Spain, 611; in Switzerland, 514. Schoolteachers were employed in elementary schools, middle schools, and high schools. The characteristics of the three samples are presented in Table 1. We note that, because we had no information on the number of schoolteachers who got access to our Internet survey through the filter of school administrators, we could not estimate response rates. The study is part of a research project funded by the Swiss National Science Foundation.

**TABLE 1:** Characteristics of the French, Spanish, and Swiss samples.

	**French sample**	**Spanish sample**	**Swiss sample**
*N*	4,395	611	514
% female	86	70	68
Age (*M* [*SD*])	44.78 (9.35)	45.98 (9.39)	44.95 (10.54)
Length of employment (*M* [*SD*])	18.31 (9.69)	16.26 (10.58)	15.91 (10.45)
% full time	87	86	47
Weekly working hours			
About 10 or less (%)	1	2	2
About 20 (%)	4	9	9
About 30 (%)	15	25	27
About 40 (%)	47	42	35
About 50 (%)	26	17	22
About 60 (%)	6	3	4
About 70 or more (%)	2	2	1
% married/sentimentally engaged	84	77	84
Number of child(ren)			
Zero (%)	20	38	29
One (%)	15	19	14
Two (%)	43	36	37
Three (%)	18	6	14
Four (%)	3	1	4
More than four (%)	1	0	1
Turnover intention ascribed to job stress (%)	18	5	10

*M, mean; SD, standard deviation. Total percentages may not equal 100 due to nearest-unit rounding. Age and length of employment are expressed in years.*

### Measures

#### Burnout

Burnout symptoms were assessed with the MBI for Educators (Dion and Tessier, 1994; Maslach et al., 2016; Gómez García et al., 2019). The MBI consists of 22 items covering the emotional exhaustion (e.g., “I feel emotionally drained from my work”), depersonalization (e.g., “I feel I treat some students as if they were impersonal objects”), and (diminished) personal accomplishment (e.g., “I deal very effectively with the problems of my students”) components of the syndrome. A 7-point rating scale was used, from 0 for “never” to 6 for “every day.” The operationalization of burnout reflected in the MBI corresponds to the currently dominant conceptualization of the syndrome. In view of Maslach et al.’s (2001) claim that burnout is a syndrome *combining* emotional exhaustion, depersonalization, and diminished personal accomplishment, we relied on a composite burnout score. Cronbach’s alphas for the MBI were 0.872, 0.893, and 0.896 in the French, Spanish, and Swiss samples, respectively.

#### Neuroticism

Neuroticism was assessed with the 12-item neuroticism subscale of the NEO-Five Factor Inventory (NEO-FFI; Rolland et al., 1998; McCrae and Costa, 2004; Aluja et al., 2005). The measure includes items such as “I often feel inferior to others.” A 5-point rating scale was employed, from 1 for “strongly disagree” to 5 for “strongly agree.” The instructions to respondents explicitly stipulated that the questions pertained to how respondents felt and behaved in general in their life. The NEO-FFI has been developed to assess neuroticism, extraversion, openness to experience, agreeableness, and conscientiousness—the “Big Five” personality traits. The NEO-FFI is an instrument of reference in personality research (McCrae and Costa, 2004). Cronbach’s alphas for the neuroticism subscale of the NEO-FFI were 0.853, 0.799, and 0.862 in the French, Spanish, and Swiss samples, respectively.

#### Sentimental Accomplishment and Familial Accomplishment

In order to assess sentimental accomplishment and familial accomplishment, we created two instruments, namely, the Sentimental Accomplishment Inventory (SAI) and the Familial Accomplishment Inventory (FAI). The SAI is intended for individuals who are engaged in a marital/sentimental relationship; the FAI is intended for individuals who have an offspring. Each measure consisted of nine items (e.g., “My relationship with my partner gives me a sense of security in my life”; “My family gives me a sense of security in life”), five of which were negatively worded (e.g., “I am experiencing or I expect to experience undesirable changes in my relationship with my partner”; “I am experiencing or I expect to experience undesirable changes in my family life”). Each item was rated using a 5-point scale, from 1 for “strongly disagree” to 5 for “strongly agree.” To design the items, we drew inspiration from existing instruments such as the Satisfaction with Life Scale (Diener et al., 1985) and the Effort-Reward Imbalance Questionnaire (Siegrist et al., 2004).

We submitted the newly developed SAI and the FAI to factor analyses to examine their latent structure. We did so with Mplus 8 (Muthén and Muthén, 1998-2017). Exploratory inspections of the SAI and FAI indicated that the nine items of each scale were reflective of three factors. We labeled these factors Esteem (e.g., “I feel valued by my partner”; “I feel valued by my family members”), Burden (e.g., “My relationship with my partner has become increasingly difficult to manage”; “My family life has become increasingly difficult to manage”), and Fulfillment (e.g., “All in all, my partner meets my expectations”; “All in all, my family life meets my expectations”). We further examined the factorial structure of the two scales based on exploratory structural equation modeling (ESEM) bifactor analysis. As noted by Marsh et al. (2014), ESEM represents “an overarching integration of the best aspects of [confirmatory factor analysis/structural equation modeling] and traditional [exploratory factor analysis]” (p. 85). Bifactor analysis is particularly useful in resolving unidimensionality versus multidimensionality issues (Rodriguez et al., 2016). We treated the items as ordinal. We relied on the weighted least squares—mean and variance adjusted—(WLSMV) estimator and used a bi-geomin rotation. One general factor and three specific factors-one for each component, namely, Esteem, Burden, and Fulfillment-were extracted in each scale. Explained Common Variance (ECV) was computed in order to examine the percentage of common variance attributable to the general factor (Rodriguez et al., 2016). Results are summarized in Table 2. For both the SAI and the FAI, our ESEM bifactor model showed an excellent fit. All items loaded strongly on the general factor (*M*_*S*__*A*__*I*_ = 0.838, *SD*_*S*__*A*__*I*_ = 0.050; *M*_*F*__*A*__*I*_ = 0.756, *SD*_*F*__*A*__*I*_ = 0.086) and more strongly on the general factor than on any of the specific factors. As per the ECV indices, the general factor accounted for more than 80% of the common variance extracted; such high percentages are suggestive of essential unidimensionality (Rodriguez et al., 2016). We further inspected the unidimensionality of the SAI and the FAI based on Mokken scaling (Table 2). We conducted our Mokken scale analyses using the Mokken package version 3.0.3 (van der Ark, 2012) in R version 4.0.3 (R Core Team, 2020). Regarding the SAI, the scale-level *H* coefficient was far above the 0.50 threshold for a “strong” scale, even with the standard error taken into account. Item-level *H*s ranged from 0.526 (standard error [*SE*] = 0.013) to 0.650 (*SE* = 0.010). The Automated Item Selection Procedure (AISP) left no item unscalable at the 0.30 threshold. Regarding the FAI, the scale-level *H* coefficient and its standard error were indicative of “moderate,” though still substantial, scalability. Item-level *H*s ranged from 0.366 (*SE* = 0.014) to 0.557 (*SE* = 0.010). Again, the AISP did not leave any item unscalable at the 0.30 threshold. On the basis of our ESEM bifactor analyses and Mokken scale analyses, we considered the SAI and the FAI unidimensional measures in subsequent analyses. Negatively worded items were reverse scored when computing SAI and FAI mean scores.

**TABLE 2:** ESEM bifactor analysis and Mokken scale analysis of the Sentimental Accomplishment Inventory and Familial Accomplishment Inventory.

	***n***	**RMSEA (90% CI)**	**CFI**	**TLI**	**WRMR**	**χ*^2^* (df)**	**ECV**	**Loevinger’s *H* (*SE*) [95% CI]**
Sentimental Accomplishment Inventory	4,586	0.019 (0.008, 0.031)	1.000	0.999	0.169	16.320 (6)	0.867	0.599 (0.010) [0.580, 0.618]
Familial Accomplishment Inventory	4,243	0.018 (0.005, 0.030)	1.000	0.999	0.179	13.806 (6)	0.803	0.480 (0.009) [0.462, 0.498]

*CI, confidence interval; ECV, explained common variance; ESEM, exploratory structural equation modeling; SE, standard error. The Sentimental Accomplishment Inventory and the Familial Accomplishment Inventory are available in Supplementary Materials 1, 2 (English, French, and Spanish versions).*

The SAI and the FAI are available in Supplementary Materials 1, 2. Cronbach’s alphas for the SAI were 0.913, 0.929, and 0.938 in the French, Spanish, and Swiss samples, respectively. Cronbach’s alphas for the FAI were 0.867, 0.907, and 0.901 in the French, Spanish, and Swiss samples, respectively. Our survey was designed so that only participants who self-identified as married/sentimentally engaged completed the SAI and only participants who declared to have at least one child completed the FAI.

#### Leisure Activities

Leisure activities were assessed based on a three-item measure (e.g., “In your life outside of work, do you have the possibility to devote yourself to hobbies or passions?”). A 5-point rating scale was employed, from 1 for “not at all” to 5 for “totally.” Cronbach’s alphas for our measure of leisure activities were 0.870, 0.904, and 0.885 in the French, Spanish, and Swiss samples, respectively.

#### Residential Satisfaction, Environmental Quality, and Security in Daily Life

Residential satisfaction was assessed with the item “Are you satisfied with where you live?”; environmental quality, with the item “Is your living environment outside of work healthy in terms of pollution control, noise levels, hygiene, etc.?”; and security in daily life, with the item “Do you feel safe where you live?” Participants responded using a 5-point rating scale, from 1 for “not at all” to 5 for “totally.” Residential satisfaction, environmental quality, and security in daily life reflect basic aspects of quality of life as conceived of by the World Health Organization (The WHOQOL Group, 1998).

#### Job Strain

Job strain was measured with a shortened version of the Effort-Reward Imbalance Questionnaire (Niedhammer et al., 2004; Fernandez-Lopez et al., 2006; Siegrist et al., 2009).^1^ Job-related efforts were assessed with four items covering workload and time pressure at work, work interruption/disturbance, responsibility in one’s job, and overtime work. Job-related rewards were assessed with six items covering performance-related esteem, social recognition, job security, organizational justice, adequacy between occupational position and education/training, and wage satisfaction. A 5-point rating scale was employed, from 1 for “strongly disagree” to 5 for “strongly agree.”

The effort-reward imbalance model defines job strain on the basis of the *ratio* of effort to reward. This definition derives from the view that it is the discrepancy between effort and reward, rather than effort or reward *per se*, that affects individuals’ well-being (Siegrist et al., 2004). Mean effort scores were divided by mean reward scores to obtain the ratio in question. Mathematically speaking, an effort-reward ratio of 1 is indicative of a perfect balance between the efforts and the rewards. A ratio <1 indicates that the rewards outweigh the efforts whereas an effort-reward ratio > 1 indicates that the efforts outweigh the rewards. Cronbach’s alphas of 0.703, 0.702, and 0.716 were obtained in the French, Spanish, and Swiss samples, respectively. The effort-reward imbalance model is a model of reference in job stress research (Wang et al., 2012; Eddy et al., 2016, 2018).

#### Illegitimate Work Tasks

Illegitimate work tasks were assessed with two items from the Bern Illegitimate Tasks Scale (BITS; Semmer et al., 2015). The first item pertained to unreasonable work tasks (“At work, I am asked to perform tasks that I think should be performed by someone else”). The second item pertained to unnecessary work tasks [“At work, I am asked to perform tasks that would not have to be performed (or could be performed with less effort) if things were organized differently”]. A 5-point rating scale was employed, from 1 for “strongly disagree” to 5 for “strongly agree.” Substantial associations have been observed between illegitimate work tasks and burnout/depression in previous studies of educational staff members (Bianchi and Schonfeld, 2018), suggesting that illegitimate work tasks constitute highly relevant stressors in that occupational area.

#### Job Autonomy, Skill Development, and Performance Feedback

Job autonomy was assessed with the item “In my job, I have the autonomy I need”; skill development, with the item “In my job, I have the opportunity to acquire knowledge and develop my skills”; and performance feedback, with the item “In my job, I am provided with constructive feedback on the quality of my work.” Participants responded using a 5-point rating scale, from 1 for “strongly disagree” to 5 for “strongly agree.” Job autonomy, skill development, and performance feedback have been considered important job resources in occupational health research (e.g., Schaufeli et al., 2009; Hakanen et al., 2019).

#### Support in Work Life and Support in Personal Life

Support in work life and support in personal life were each assessed with a single-item measure rated on a 7-point scale (from 1 for “not at all” to 7 for “very strongly”): “To what extent can you rely on the people around you (e.g., friends, colleagues, and family members) when problems arise in your professional life?”; “To what extent can you rely on the people around you (e.g., friends, colleagues, and family members) when problems arise in your personal life?”. Single-item measures of social support have proved effective in a number of studies (e.g., Friedman et al., 2017; Bianchi and Schonfeld, 2020). Social support is considered an important resource to successfully deal with (job) stressors (van der Doef and Maes, 1999; Halbesleben, 2006).

#### Work–Non-work Conflict and Non-work–Work Conflict

Work–non-work conflict and non-work–work conflict were each assessed with a single-item measure rated on a 7-point scale (from 1 for “no, not at all” to 7 for “yes, very clearly”): “Do you feel that your professional life has a negative impact on your personal life?”; “Do you feel that your personal life has a negative impact on your professional life?”. Similar items have been used in past studies of between-domain interferences and burnout (e.g., Blom et al., 2014).

#### Sociodemographic and Additional Occupational/Health Items

Participants provided information about their sex, age, length of employment, full-time versus part-time position, weekly working hours, marital/sentimental status, number of child(ren), turnover intention ascribed to job stress (“Are you considering leaving your job due to excessive work stress?”; response options: “yes”; “no”; “I don’t know”), and physical condition (“Are you satisfied with your physical condition?”; from 1 for “not at all” to 7 for “totally”). Physical activity and general health status have been associated with burnout in many studies (e.g., Toker and Biron, 2012; Bianchi and Schonfeld, 2018).

### Data Analyses

We analyzed our data based on correlational analysis, multiple regression analysis, and relative weight analysis using IBM SPSS Statistics v20 and RWA Web (Tonidandel and LeBreton, 2015). Relative weight analysis allows the investigator to determine “the contribution a variable makes to the prediction of a criterion variable by itself and in combination with other predictor variables” (Tonidandel and LeBreton, 2011, p. 2). As underlined by Tonidandel and LeBreton (2015), “[t]he typical indices produced by regression are useful but do not accurately partition variance among correlated predictors, whereas relative weight analysis is properly suited for this function” (p. 215). The use of relative weight analysis responded to a key objective of our study, which was to establish a hierarchy among our predictors of interest.

Burnout constituted our dependent variable. Our 22 predicting variables fell into four categories: (a) individual-level factors (four variables), which included neuroticism, sex, age, and physical condition; (b) work factors (eight variables), which included job strain, unreasonable work tasks, unnecessary work tasks, weekly working hours, job autonomy, skill development, performance feedback, and support in work life; (c) non-work factors (eight variables), which included sentimental accomplishment, familial accomplishment, number of child(ren), leisure activities, residential satisfaction, environmental quality, security in daily life, and support in personal life; and (d) intersecting factors (two variables), which included work–non-work conflict and non-work–work conflict. Work–non-work conflict and non-work–work conflict were considered intersecting, rather than work or non-work, factors because of their *relational* nature. Work–non-work conflict, for instance, does not necessarily imply that the job is in itself stressogenic. Professional life can affect personal life negatively by being strongly immersive, invested-in, and time- and energy-consuming. The same line of reasoning can be applied to non-work–work conflict.

The use of three different samples enabled us to assess the replicability of our findings within a single study. Such a framework is helpful in terms of external validity.

## Results

### Correlational Analyses

Zero-order correlations among the main study variables are available in Supplementary Material 3. In the French sample, burnout was significantly associated with all the other variables except sex. Burnout correlated on average 0.233 with work factors (*SD* = 0.101). In the Spanish sample, burnout was significantly associated with all the other variables except sex and age. Burnout correlated on average 0.271 with work factors (*SD* = 0.099). In the Swiss sample, burnout was significantly associated with all the other variables except sex, age, and weekly working hours. Burnout correlated on average 0.284 with work factors (*SD* = 0.114). In each of the three samples, burnout correlated primarily with neuroticism, with *r*s ranging from 0.552 to 0.642 (all *p*s < 0.001). When corrected for attenuation, the burnout-neuroticism correlation reached 0.640 in the French sample, 0.665 in the Spanish sample, and 0.731 in the Swiss sample.

### Multiple Regression Analyses

The results of our multiple regression analyses are summarized in Table 3. The model under scrutiny explained about 45, 43, and 60% of the variance in burnout in the French, Spanish, and Swiss samples, respectively. In the French sample, 14 of the 22 independent variables were predictive of burnout. Burnout showed positive associations with neuroticism, sentimental accomplishment, job strain, unreasonable work tasks, work–non-work conflict, non-work–work conflict, sex, and age and negative associations with leisure activities, security in daily life, weekly working hours, job autonomy, skill development, and support in work life. In the Spanish sample, six of the 22 independent variables were predictive of burnout. Burnout was positively predicted by neuroticism, job strain, and work–non-work conflict and negatively predicted by leisure activities, security in daily life, and skill development. In the Swiss sample, seven of the 22 independent variables were predictive of burnout. Burnout was associated positively with neuroticism, job strain, support in personal life, work–non-work conflict, and age and negatively with security in daily life and skill development. Neuroticism, security in daily life, job strain, skill development, and work–non-work conflict were thus linked to burnout in all three samples. No variance inflation factor exceeded 2.652 and the average variance inflation factor was close to 1 in all samples, suggesting that multicollinearity was not an issue in the tested model (Field, 2018).

**TABLE 3:** Summary of multiple regression analyses pertaining to the prediction of burnout—French, Spanish, and Swiss samples.

	**French sample (*n* = 3,118)**			**Spanish sample (*n* = 347)**			**Swiss sample (*n* = 323)**		
**Predictors**	**β**	***p***	**VIF**	**β**	***p***	**VIF**	**β**	***p***	**VIF**
**Individual-level factors**									
Neuroticism	0.373	0.000	1.556	0.318	0.000	1.745	0.409	0.000	1.953
Sex	0.091	0.000	1.052	0.048	0.253	1.079	0.032	0.432	1.320
Age	0.051	0.001	1.192	0.049	0.294	1.292	0.137	0.001	1.289
Physical condition	–0.004	0.773	1.325	0.055	0.231	1.291	0.009	0.831	1.350
**Work factors**									
Job strain	0.123	0.000	1.712	0.164	0.003	1.775	0.140	0.006	2.053
Unreasonable work tasks	0.051	0.002	1.499	–0.007	0.898	1.610	0.048	0.274	1.516
Unnecessary work tasks	0.002	0.911	1.452	0.067	0.192	1.592	–0.010	0.818	1.513
Weekly working hours	–0.091	0.000	1.215	0.000	0.994	1.221	–0.046	0.297	1.531
Job autonomy	–0.048	0.001	1.245	–0.006	0.896	1.417	0.041	0.336	1.417
Skill development	–0.082	0.000	1.209	–0.107	0.025	1.362	–0.159	0.000	1.218
Performance feedback	–0.020	0.167	1.148	–0.016	0.719	1.228	–0.080	0.055	1.373
Support in work life	–0.066	0.001	2.188	0.044	0.456	2.047	–0.102	0.077	2.652
**Non-work factors**									
Sentimental accomplishment	0.056	0.001	1.555	0.079	0.187	2.170	0.052	0.285	1.905
Familial accomplishment	–0.004	0.787	1.501	–0.074	0.260	2.586	0.053	0.278	1.873
Number of child(ren)	–0.016	0.257	1.087	–0.019	0.647	1.060	–0.065	0.087	1.149
Leisure activities	–0.042	0.013	1.639	–0.163	0.001	1.492	0.044	0.370	1.924
Residential satisfaction	0.010	0.539	1.422	0.044	0.402	1.643	–0.071	0.140	1.810
Environmental quality	–0.016	0.295	1.285	–0.054	0.253	1.361	–0.082	0.057	1.454
Security in daily life	–0.034	0.023	1.302	–0.106	0.028	1.386	–0.111	0.018	1.727
Support in personal life	–0.005	0.797	2.361	0.026	0.655	2.051	0.114	0.047	2.593
**Intersecting factors**									
Work–non-work conflict	0.208	0.000	1.664	0.170	0.001	1.554	0.240	0.000	2.155
Non-work–work conflict	0.031	0.040	1.258	0.003	0.950	1.596	0.065	0.139	1.519
Adjusted *R*^2^		0.447			0.426			0.596	

*VIF, variance inflation factor. Only participants who reported to be married/sentimentally engaged and to have at least one child were included in these analyses.*

### Relative Weight Analyses

The results of our relative weight analyses are summarized in Table 4. Personality trait neuroticism was found to be the best predictor of burnout in all three samples. Neuroticism alone represented about 28–34% of the explanatory power the model under consideration. The second most important predictor of burnout was an intersecting factor, namely, work-non–work conflict (about 15–18% of the explanatory power). The third rank was occupied by a work-situated factor, namely, job strain (about 10–12% of the explanatory power).

**TABLE 4:** Summary of relative weight analyses pertaining to the prediction of burnout—French, Spanish, and Swiss samples.

	**French sample (*n* = 3,118)**			**Spanish sample (*n* = 347)**			**Swiss sample (*n* = 323)**		
**Predictors**	**Raw weights**	**95% CI^a^**	**Rescaled weights^b^**	**Raw weights**	**95% CI^a^**	**Rescaled weights^b^**	**Raw weights**	**95% CI^a^**	**Rescaled weights^b^**
**Individual-level factors**									
Neuroticism	0.149	0.132, 0.167	33.520	0.134	0.093, 0.187	29.146	0.170	0.132, 0.221	28.138
Sex	0.004	0.002, 0.008	0.862	0.001	0.000, 0.004	0.204	0.002	0.000, 0.006	0.312
Age	0.002	0.001, 0.003	0.412	0.001	0.000, 0.002	0.268	0.006	0.002, 0.018	0.966
Physical condition	0.011	0.007, 0.016	2.497	0.010	0.004, 0.025	2.166	0.025	0.010, 0.049	4.106
**Work factors**									
Job strain	0.045	0.036, 0.056	10.142	0.057	0.031, 0.091	12.349	0.058	0.035, 0.086	9.684
Unreasonable work tasks	0.017	0.011, 0.023	3.761	0.007	0.003, 0.014	1.433	0.012	0.004, 0.026	1.917
Unnecessary work tasks	0.008	0.005, 0.013	1.869	0.012	0.003, 0.028	2.542	0.008	0.003, 0.019	1.260
Weekly working hours	0.004	0.002, 0.007	0.923	0.006	0.002, 0.019	1.214	0.003	0.001, 0.004	0.449
Job autonomy	0.020	0.013, 0.028	4.472	0.023	0.009, 0.045	5.022	0.022	0.007, 0.046	3.688
Skill development	0.026	0.019, 0.035	5.946	0.036	0.013, 0.071	7.816	0.029	0.010, 0.055	4.758
Performance feedback	0.008	0.004, 0.013	1.841	0.004	0.002, 0.014	0.951	0.016	0.004, 0.034	2.604
Support in work life	0.015	0.010, 0.021	3.338	0.004	0.001, 0.010	0.789	0.009	0.003, 0.021	1.536
**Non-work factors**									
Sentimental accomplishment	0.000	0.000, 0.002	0.108	0.003	0.000, 0.013	0.554	0.005	0.001, 0.016	0.852
Familial accomplishment	0.000	0.000, 0.002	0.069	0.001	0.000, 0.006	0.178	0.001	0.000, 0.007	0.234
Number of child(ren)	0.006	0.003, 0.011	1.445	0.002	0.001, 0.008	0.409	0.007	0.001, 0.021	1.086
Leisure activities	0.022	0.016, 0.029	4.851	0.042	0.018, 0.077	9.138	0.029	0.016, 0.049	4.869
Residential satisfaction	0.002	0.001, 0.004	0.371	0.002	0.001, 0.003	0.412	0.021	0.006, 0.051	3.530
Environmental quality	0.002	0.000, 0.004	0.356	0.014	0.003, 0.037	2.966	0.019	0.005, 0.042	3.167
Security in daily life	0.007	0.003, 0.012	1.559	0.020	0.006, 0.044	4.442	0.043	0.019, 0.074	7.049
Support in personal life	0.010	0.007, 0.015	2.293	0.009	0.003, 0.024	1.952	0.006	0.002, 0.014	0.979
**Intersecting factors**									
Work–non-work conflict	0.081	0.070, 0.093	18.284	0.067	0.034, 0.110	14.569	0.100	0.071, 0.136	16.553
Non-work–work conflict	0.005	0.002, 0.009	1.083	0.007	0.002, 0.022	1.480	0.014	0.004, 0.032	2.262

*Only participants who reported to be married/sentimentally engaged and to have at least one child were included in these analyses. ^*a*^95% confidence interval around the raw weights. A 10,000-iteration bootstrap was performed. ^*b*^Rescaled weights are indicative of the percentage of variance explained by each predictor. Total percentages may not equal 100 due to rounding.*

Considered together, individual-level factors represented about 32–37% of the explanatory power of our model across the three samples; work factors, about 26–32%; non-work factors, about 11–22%; and intersecting factors, about 16–19%. Factors other than work factors represented about 68–74% of the explanatory power of our model.

## Discussion

It has long been asserted that burnout is explained by occupational-context factors rather than individual-level (e.g., personality) and non-work (or general) factors (e.g., Maslach and Leiter, 2010; Shanafelt et al., 2017). We evaluated the validity of this view by examining the links between burnout and a total of 22 work-situated, work-unrelated, dispositional, and intersecting (e.g., work–non-work conflict) variables in three different schoolteacher samples from three different countries—France, Spain, and Switzerland. We used relative weight analysis for hierarchizing the importance of the predictors under examination (Tonidandel and LeBreton, 2015). Neuroticism, job strain, skill development, security in daily life, and work-non–work conflict were consistently associated with burnout across the three samples. Sample-specific predictors of burnout included sex, age, unreasonable work tasks, weekly working hours, job autonomy, support in work life, sentimental accomplishment, leisure activities, support in personal life, and non-work–work conflict. Our findings are supportive of the multilevel determinants of workers’ mental health model (Marchand et al., 2015), which predicts that work, non-work and individual factors contribute to explaining workers’ mental health.

In all samples, burnout was found to be primarily accounted for by personality trait neuroticism. Importantly, the link between neuroticism and burnout was largely independent of the levels of the other predictors. Our results are consistent with those of recent studies having employed relative weight analysis to examine the “job-relatedness” of burnout’s nomological network (Bianchi, 2018; Bianchi et al., 2018a), as well as with the strong meta-correlations documented between neuroticism and burnout (Alarcon et al., 2009; Swider and Zimmerman, 2010; You et al., 2015). Our results are also in keeping with the observation that neuroticism is a risk factor for depression (Kotov et al., 2010; Hakulinen et al., 2015; Jeronimus et al., 2016), a condition with which burnout overlaps (Bianchi et al., 2020). By contrast, our findings do not support the view that burnout is first and foremost related to occupational-context factors (e.g., Maslach and Leiter, 2010; Shanafelt et al., 2017).

The second-best predictor of burnout in the three samples was work–non-work conflict—an intersecting factor. This result is consistent with those of a number of studies that found an association between work–non-work conflict and burnout (Marchand et al., 2015; Nohe et al., 2015; Verweij et al., 2017). Interestingly, our finding suggests that the (dis)harmony between work life and life outside of work might be at least as important as job characteristics for the prediction of burnout. From an interventional standpoint, focusing on how burned out individuals coordinate and prioritize the activities related to the various domains of their life might therefore be particularly fruitful.

Although their weight was not as important as generally presumed, several work factors were associated with burnout. Job strain and skill development, in particular, were consistently linked to burnout across the three samples. These results are in line with a large body of findings (e.g., Schaufeli et al., 2009; Verweij et al., 2017), including findings pertaining specifically to teachers (e.g., Santavirta et al., 2007; Bianchi, 2018). It is worth noting, however, that job strain represented only 10–12% of the explanatory power of the examined model despite the involvement of key job-related characteristics in its assessment (workload and time pressure at work, work interruption/disturbance, responsibility in one’s job, overtime work, performance-related esteem, social recognition, job security, organizational justice, adequacy between occupational position and education/training, and wage satisfaction). In the largest of our three samples—the French sample, unreasonable work tasks, weekly working hours, job autonomy, and support in work life also appeared to play a role in predicting burnout, in keeping with previously reported findings (e.g., Schaufeli et al., 2009; Marchand et al., 2015; Bianchi and Schonfeld, 2018).

Burnout was also found to be associated with several non-work factors. Security in daily life predicted burnout negatively in all three samples and represented up to 7% of the explanatory power of the examined model—in the Swiss sample. Leisure activities showed negative associations with burnout in two of our three samples, representing up to 9% of the explanatory power of the examined model—in the Spanish sample. The importance of off-job activities for work-related fatigue and engagement has been highlighted in several past studies (ten Brummelhuis and Bakker, 2012; Garrick et al., 2018). Our results confirm that non-work/general factors should not be overlooked when addressing the issue of burnout (Verweij et al., 2017).

### Implications of Our Findings for Burnout’s Conceptual Status

Our findings have implications for the conceptual status of burnout. The idea that burnout is mainly linked to occupational-context factors has been used to justify the conceptual distinctiveness of burnout, notably vis-à-vis depression (e.g., Maslach et al., 2001). As a reminder, in this study, work factors represented 26–32% of the explanatory power of the examined model whereas individual-level, non-work, and intersecting factors totalized 68–74% of that power. Such results suggest that “job-relatedness” may not constitute a solid basis for characterizing or singularizing the burnout phenomenon. Burnout is indeed partly explained by work factors, but conditions such as depression are also predicted in part by work factors (Madsen et al., 2017). Our results dovetail with a growing body of findings pertaining to the link between burnout and job variables. For instance, Bianchi and Brisson (2019) found that many individuals with burnout symptoms (about 50% in their sample) do not ascribe their symptoms to their job (see also Bianchi et al., 2018b). In a study by Leiter and Maslach (2004), the supposedly most critical organizational antecedents of burnout—workload, control, reward, community, fairness, and values—explained less than 7% of the variance in the syndrome on average (*N* = 6,815). On a different note, evidence for a link between burnout and “objective” job performance (e.g., formally recorded medical errors in the case of physician burnout) is, on balance, disquietingly weak (Taris, 2006; Orton et al., 2012; Tyssen, 2018; Bianchi et al., 2019; Mirkovic and Bianchi, 2019). All in all, our results further question the view that burnout’s nomological network is primarily job-related.

### Study Limitations and Avenues for Future Research

Our study has at least six limitations. First, our study had a cross-sectional design. Causal inferences regarding the links between our variables of interest should therefore be avoided until longitudinal research in conducted. This being mentioned, longitudinal research pertaining to neuroticism indicates that neuroticism is a vulnerability factor for, rather than an outcome of, general distress (Williams et al., 2020). Neuroticism can thus be expected to predict burnout rather than the other way around. Second, even if the replication of our main finding (i.e., the primary role of neuroticism in predicting burnout) across three different samples recruited in three different countries suggests that this finding has some degree of generalizability, it remains that we relied on convenience sampling. Such a sampling method is susceptible to selection bias. Studies relying on probability sampling would be useful. Third, with the aim of reducing respondent burden, we assessed several of our variables of interest (e.g., social support) using single-item measures. Single-item measures can be used effectively to assess many constructs, notably global or unidimensional constructs (Bowling, 2005; Fisher et al., 2016), and there is evidence that the problem of the reliability of single-item measures has been overstated (Friedman et al., 2017; McCrae and Mõttus, 2019; Mõttus and Rozgonjuk, 2019). The use of multiple-item measures, however, is generally preferable. Fourth, our study samples were predominantly female, consistent with the fact that most educational staff members are women in countries such as France, Spain, and Switzerland (e.g., Ministère de l’Éducation Nationale, 2017). Reassuringly, however, sex correlated weakly, when at all, with our other variables of interest (Supplementary Material 3), including variables such as work–non-work and non-work–work conflicts (for discussions of the relationships between work–non-work and non-work–work conflicts and sex, see Shockley et al., 2017; see also Zhou et al., 2018). Fifth, our study involved only one occupational group, namely, schoolteachers. Studies of other occupational groups could be informative. Sixth, although we covered a relatively large range of factors in this study, additional variables could have been examined. Most notably, it would have been a plus if a greater number of personality traits had been considered. For instance, borderline personality traits have shown strong associations with burnout (Bianchi et al., 2018b; Brenning et al., 2020). Additional personality traits of the Big Five, such as extraversion and conscientiousness, would have also been worth investigating (Kotov et al., 2010; Swider and Zimmerman, 2010; Watson et al., 2019). It is probable that the link between personality and burnout, though pre-eminent in our findings, is still underestimated in this study.

Because our variables were assessed with self-reported measures, it might be contended that our findings are threatened by monomethod bias (Podsakoff et al., 2003). Without even underlining that the problem of monomethod bias has been overstated in psychological research (Spector, 2006), such a contention is of limited relevance to the present study. Indeed, even if the action of common method variance were operant in our study, there is no reason to consider that such an action would more strongly affect, for instance, the link between burnout and neuroticism than the link between burnout and job strain (or between burnout and other work-situated factors). In other words, the action of common method variance is unlikely to bear on the predictor hierarchy established in the study and, subsequently, on the study conclusions.

We note that our French sample was considerably larger than our Spanish and Swiss samples. Consequently, our ability to identify “statistically significant” associations was much greater in the French sample than in the other two samples. In keeping with general statistical guidelines, we recommend that the study results be interpreted in the light of effect sizes (Cumming, 2014).

## Conclusion

This relative weight analytic study suggests that (a) burnout is primarily associated with personality trait neuroticism, (b) interferences between the work and non-work domains are at least as important as “purely occupational” factors in accounting for burnout, and (c) general factors such as security in daily life are relevant predictors of burnout. It has long been speculated that burnout had little to do with personal features and non-work factors and was essentially linked to poor working conditions (Maslach and Leiter, 1997). While the adverse effects of poor working conditions on health cannot be overstated, our study invites investigators to more systematically consider dispositional characteristics and non-occupational factors when addressing the issue of burnout. Failing to do so might obscure our understanding of burnout and impede our ability to treat and prevent the syndrome. It is well established that personality traits, most notably neuroticism, can be changed as a result of therapy (Roberts et al., 2017; Bleidorn et al., 2019; Hanley et al., 2019). Therapeutic action targeting neuroticism might help us combat burnout more effectively. To conclude, we recommend that the determinants of burnout be approached relationally and through a multiscale lens (e.g., individual, organizational, social). Acting as if the answers to job stress questions lay either exclusively in individuals or exclusively in individuals’ environment (e.g., organizational environment and social environment) may considerably undermine our efforts to promote occupational health.

## Data Availability Statement

The raw data supporting the conclusions of this article will be made available by the authors, without undue reservation.

## Ethics Statement

Ethical review and approval was not required for the study on human participants in accordance with the local legislation and institutional requirements. The patients/participants provided their written informed consent to participate in this study.

## Author Contributions

RB developed the study concept, conducted the data collection in France and Switzerland, performed the data analysis, and drafted the manuscript. GM-G conducted the data collection in Spain. All authors took part in result interpretation. All authors reviewed and edited several versions of the manuscript and provided critical revisions. All authors approved the final version of the manuscript for submission.

## Conflict of Interest

The authors declare that the research was conducted in the absence of any commercial or financial relationships that could be construed as a potential conflict of interest.

## Footnotes

1 https://www.uniklinik-duesseldorf.de/patienten-besucher/klinikeninstitutezentren/
