# Supplementary material for: Is Burnout Primarily Linked to Work-Situated Factors? A Relative Weight Analytic Study
Source: Front Psychol. 2021 Jan 13;11:623912. doi: 10.3389/fpsyg.2020.623912 (PMC7838215; doi:10.3389/fpsyg.2020.623912)
Supplement: Supplementary file 1 [file Data_Sheet_1.PDF]

Supplementary Material 1.

SENTIMENTAL ACCOMPLISHMENT INVENTORY

1. *I receive the respect I deserve from my partner.*

   Je reçois le respect que je mérite de la part de mon/ma partenaire.

   Recibo el respeto que merezco de mi pareja.

2. *I am experiencing or I expect to experience undesirable changes in my relationship with my partner.**

   Je vis ou je m'attends à vivre des changements indésirables dans ma relation avec mon/ma partenaire.

   Estoy experimentando o espero experimentar cambios indeseables en mi relación con mi pareja.

3. *My partner treats me unfairly.**

   Mon/Ma partenaire me traite de manière injuste.

   Mi pareja me trata injustamente.

4. *I feel that there is no future with my partner.**

   Je sens qu'il n'y a pas d'avenir avec mon/ma partenaire.

   Siento que no hay futuro con mi pareja.

5. *I have a tormented and complicated relationship with my partner.**

   J'ai une relation tourmentée et compliquée avec mon/ma partenaire.

   Tengo una relación atormentada y complicada con mi pareja.

6. *All in all, my partner meets my expectations.*

   Tout bien considéré, mon/ma partenaire satisfait mes attentes.

   En general, mi pareja cumple con mis expectativas.

7. *My relationship with my partner gives me a sense of security in my life.*

   Ma relation avec mon/ma partenaire me donne un sentiment de sécurité dans ma vie.

   Mi relación con mi pareja me da una sensación de seguridad en mi vida.

8. *My relationship with my partner has become increasingly difficult to manage.**

   Ma relation avec mon/ma partenaire est devenue de plus en plus difficile à gérer.

   Mi relación con mi pareja se ha vuelto cada vez más difícil de manejar.

9. *I feel valued by my partner.*

   Je me sens valorisé(e) par mon/ma partenaire.

   Me siento valorado por mi pareja.

* Reverse scoring required.
