# Supplementary material for: Is Burnout Primarily Linked to Work-Situated Factors? A Relative Weight Analytic Study
Source: Front Psychol. 2021 Jan 13;11:623912. doi: 10.3389/fpsyg.2020.623912 (PMC7838215; doi:10.3389/fpsyg.2020.623912)
Supplement: Supplementary file 2 [file Data_Sheet_2.PDF]

Supplementary Material 2.

FAMILIAL ACCOMPLISHMENT INVENTORY

1. *My family members give me the respect I deserve.*

   Les membres de ma famille m'accordent le respect que je mérite.

   Los miembros de mi familia me dan el respeto que merezco.

2. *I am experiencing or I expect to experience undesirable changes in my family life.**

   Je vis ou je m'attends à vivre des changements indésirables dans ma vie familiale.

   Estoy experimentando o espero experimentar cambios indeseables en mi vida familiar.

3. *My family members treat me unfairly.**

   Les membres de ma famille me traitent de manière injuste.

   Los miembros de mi familia me tratan injustamente.

4. *My family life is complicated and I think it will remain so.**

   Ma vie familiale est compliquée et je crois qu'elle le restera.

   Mi vida familiar es complicada y creo que seguirá siéndolo.

5. *I am often in conflict with family members.**

   Je suis souvent en conflit avec des membres de ma famille.

   A menudo estoy en conflicto con miembros de mi familia.

6. *All in all, my family life meets my expectations.*

   Tout bien considéré, ma vie de famille satisfait mes attentes.

   En general, mi vida familiar satisface mis expectativas.

7. *My family gives me a sense of security in life.*

   Ma famille me donne un sentiment de sécurité dans la vie.

   Mi familia me da una sensación de seguridad en la vida.

8. *My family life has become increasingly difficult to manage.**

   Ma vie de famille est devenue de plus en plus difficile à gérer.

   Mi vida familiar se ha vuelto cada vez más difícil de manejar.

9. *I feel valued by my family members.*

   Je me sens valorisé(e) par les membres de ma famille.

   Me siento valorado por los miembros de mi familia.

* Reverse scoring required.
