# Supplementary material for: Is Burnout Primarily Linked to Work-Situated Factors? A Relative Weight Analytic Study
Source: Front Psychol. 2021 Jan 13;11:623912. doi: 10.3389/fpsyg.2020.623912 (PMC7838215; doi:10.3389/fpsyg.2020.623912)
Supplement: Supplementary file 3 [file Data_Sheet_3.PDF]

Supplementary Material 3a. Zero-order correlations among the main study variables in the French sample (*N* = 4,395).

		M	*SD*	2.	3.	4.	5.	6.	7.	8.	9.	10.	11.	12.	13.	14.	15.	16.	17.	18.	19.	20.	21.	22.	23.
1.	Burnout	1.831	0.897	.552	-.115	-.226	-.121	-.306	-.108	-.081	-.187	-.250	.400	.252	.208	.039	-.265	-.259	-.179	-.258	-.226	.477	.130	*.022*	-.051
2.	Neuroticism	2.693	0.793	—	-.219	-.336	-.107	-.338	-.137	-.070	-.213	-.345	.336	.162	.129	.078	-.190	-.175	-.116	-.262	-.267	.452	.193	-.107	-.100
3.	SA	4.402	0.763		—	.468	*-.025*	.132	.149	.073	.154	.122	-.044	-.048	*-.002*	*-.014*	*.024*	.064	*.006*	.317	.413	-.111	-.382	*-.021*	-.081
4.	FA	4.327	0.686			—	.072	.202	.227	.097	.237	.237	-.146	-.096	-.057	*.020*	.088	.083	.047	.331	.382	-.204	-.338	*.015*	.043
5.	Number of child(ren)	1.709	1.112				—	*.007*	.152	.122	.083	.056	-.045	*-.011*	-.041	-.040	*.009*	*-.011*	-.057	.038	*.005*	-.089	*-.023*	*.025*	.415
6.	Leisure activities	3.073	1.033					—	.130	.065	.149	.407	-.354	-.156	-.139	-.292	.183	.102	.088	.241	.225	-.457	-.071	.136	.109
7.	Residential satisfaction	6.100	1.252						—	.486	.424	.129	-.074	*-.004*	*-.026*	-.039	.036	.032	*-.025*	.113	.118	-.126	-.116	-.052	.131
8.	Environmental quality	5.980	1.394							—	.331	.084	-.049	*-.024*	-.040	*-.018*	.051	*.005*	*-.020*	.055	.063	-.062	-.072	-.046	*.029*
9.	Security in daily life	6.420	0.955								—	.176	-.155	-.057	-.061	-.058	.154	.080	*-.020*	.147	.170	-.147	-.130	-.046	*.024*
10.	Physical condition	4.280	1.632									—	-.216	-.108	-.100	-.121	.143	.081	.109	.178	.183	-.270	-.095	.058	*.007*
11.	Job strain	1.305	0.528										—	.387	.351	.269	-.327	-.187	-.235	-.200	-.140	.462	*.009*	-.065	-.085
12.	Unreasonable work tasks	3.590	1.323											—	.515	.128	-.214	-.121	-.147	-.074	-.064	.256	.039	*-.008*	*.004*
13.	Unnecessary work tasks	3.900	1.169												—	.123	-.205	-.071	-.166	-.081	-.062	.247	*.005*	*.018*	*-.003*
14.	Weekly working hours	41.907	9.851													—	-.077	.090	*.007*	*-.028*	-.033	.226	-.056	*-.021*	*-.024*
15.	Job autonomy	3.800	1.001														—	.280	.132	.122	.117	-.223	*.018*	*.001*	-.075
16.	Skill development	3.460	1.246															—	.272	.152	.124	-.146	*-.030*	*-.008*	-.054
17.	Performance feedback	1.800	1.013																—	.123	.074	-.147	*-.002*	*.012*	-.030
18.	Support in work life	5.190	1.660																	—	.730	-.210	-.156	-.037	-.075
19.	Support in personal life	5.510	1.556																		—	-.165	-.195	-.054	-.147
20.	Work-nonwork conflict	4.170	1.986																			—	.133	-.072	-.158
21.	Nonwork-work conflict	2.080	1.471																				—	*.009*	-.065
22.	Sex	0.140	0.349																					—	.105
23.	Age	44.775	9.349																						—

Notes. SA: sentimental accomplishment; FA: familial accomplishment. Correlations pertaining to sentimental accomplishment involved only 3,682 participants (about 16% of the French participants were not married/sentimentally engaged); correlations pertaining to familial accomplishment involved only 3,501 participants (about 20% of the French participants were childless). Nonsignificant correlations ($p > .05$) are italicized.

Supplementary Material 3b. Zero-order correlations among the main study variables in the Spanish sample (*N* = 611).

		M	*SD*	2.	3.	4.	5.	6.	7.	8.	9.	10.	11.	12.	13.	14.	15.	16.	17.	18.	19.	20.	21.	22.	23.
1.	Burnout	1.254	0.860	.562	-.139	-.277	-.087	-.349	-.157	-.203	-.251	-.195	.422	.288	.253	.116	-.320	-.365	-.225	-.179	-.220	.486	.211	*.058*	*.006*
2.	Neuroticism	2.228	0.691	—	-.256	-.414	-.082	-.311	-.228	-.241	-.345	-.311	.389	.276	.232	*.033*	-.279	-.242	-.180	-.221	-.301	.410	.274	*.011*	*-.069*
3.	SA	4.508	0.717		—	.691	*-.079*	.131	.212	*.085*	.246	.147	*-.064*	-.100	*-.036*	*.003*	*.068*	.152	*.065*	.376	.309	-.202	-.426	*-.009*	*-.022*
4.	FA	4.430	0.688			—	*.059*	.212	.353	.201	.334	.241	-.174	-.115	*-.084*	*-.031*	.101	*.094*	.110	.389	.382	-.380	-.536	*.008*	*.032*
5.	Number of child(ren)	1.129	1.033				—	-.128	.133	.104	*.052*	*-.049*	*-.051*	*-.052*	*-.025*	.091	.136	*.043*	.092	*.040*	*.046*	-.125	*.016*	*-.033*	.424
6.	Leisure activities	3.630	1.038					—	.129	.108	.151	.320	-.418	-.184	-.166	-.252	.181	.177	.160	.145	.169	-.323	-.212	*.073*	*.027*
7.	Residential satisfaction	5.880	1.271						—	.382	.426	.236	-.153	-.079	*-.055*	*.009*	.197	.133	*.035*	.292	.221	-.122	-.199	*-.061*	.090
8.	Environmental quality	5.770	1.237							—	.351	.225	-.203	-.111	*-.076*	*-.015*	.219	.156	*.057*	.229	.226	-.117	-.140	*-.046*	*.002*
9.	Security in daily life	6.310	0.963								—	.214	-.231	-.120	-.081	*-.064*	.217	.177	.119	.200	.242	-.160	-.123	*-.079*	*-.048*
10.	Physical condition	4.660	1.642									—	-.233	-.121	-.150	-.118	.141	.111	*.078*	.136	*.061*	-.210	-.176	*.020*	*-.014*
11.	Job strain	0.878	0.380										—	.482	.433	.282	-.449	-.275	-.306	-.200	-.203	.416	.121	*-.028*	*-.022*
12.	Unreasonable work tasks	2.620	1.422											—	.523	.203	-.323	-.180	-.170	-.109	-.118	.317	.096	*.034*	*.028*
13.	Unnecessary work tasks	3.440	1.290												—	.197	-.344	-.193	-.239	-.145	-.160	.234	*.038*	*.078*	*.026*
14.	Weekly working hours	38.085	11.004													—	-.149	*-.028*	-.089	*-.015*	*-.010*	.143	*-.007*	*-.006*	.162
15.	Job autonomy	3.960	1.022														—	.391	.294	.200	.205	-.301	-.092	*-.078*	*.010*
16.	Skill development	4.120	1.039															—	.338	.206	.237	-.309	*-.078*	-.082	*-.051*
17.	Performance feedback	3.170	1.218																—	.193	.198	-.210	-.105	*-.049*	*.025*
18.	Support in work life	5.800	1.510																	—	.653	-.222	-.200	*-.052*	-.141
19.	Support in personal life	5.940	1.266																		—	-.171	-.187	*-.068*	-.190
20.	Work-nonwork conflict	2.270	1.740																			—	.303	*.014*	-.137
21.	Nonwork-work conflict	1.690	1.253																				—	*-.014*	*-.067*
22.	Sex	0.300	0.458																					—	.091
23.	Age	45.980	9.393																						—

Notes. SA: sentimental accomplishment; FA: familial accomplishment. Correlations pertaining to sentimental accomplishment involved only 473 participants (about 23% of the Spanish participants were not married/sentimentally engaged); correlations pertaining to familial accomplishment involved only 377 participants (about 38% of the Spanish participants were childless). Nonsignificant correlations ($p > .05$) are italicized.

Supplementary Material 3c. Zero-order correlations among the main study variables in the Swiss sample (*N* = 514).

		M	*SD*	2.	3.	4.	5.	6.	7.	8.	9.	10.	11.	12.	13.	14.	15.	16.	17.	18.	19.	20.	21.	22.	23.
1.	Burnout	1.576	0.911	.642	-.183	-.286	-.111	-.394	-.296	-.231	-.389	-.314	.493	.280	.244	*.076*	-.301	-.299	-.264	-.314	-.247	.576	.246	*-.043*	*.033*
2.	Neuroticism	2.455	0.797	—	-.259	-.362	-.099	-.397	-.238	-.121	-.371	-.339	.382	.238	.240	*.014*	-.276	-.165	-.218	-.289	-.272	.516	.289	-.122	*-.082*
3.	SA	4.432	0.829		—	.575	-.119	.235	.247	.145	.308	.113	-.188	*-.037*	*-.009*	*-.015*	.177	*.091*	*.017*	.394	.403	-.156	-.435	*.001*	-.132
4.	FA	4.307	0.777			—	*.027*	.235	.285	.229	.384	.235	-.252	-.178	-.116	*-.031*	.244	.194	.120	.455	.471	-.217	-.364	*-.039*	*-.093*
5.	Number of child(ren)	1.547	1.236				—	*-.055*	*.078*	*.061*	*.027*	*.004*	*.049*	*-.070*	*.031*	*-.061*	-.129	*-.016*	*.005*	-.096	*-.040*	*-.010*	*-.041*	.119	.502
6.	Leisure activities	3.503	1.061					—	.245	.200	.278	.375	-.496	-.234	-.189	-.252	.247	.111	.143	.247	.184	-.504	-.179	.136	*-.029*
7.	Residential satisfaction	6.099	1.217						—	.524	.422	.227	-.183	-.104	*-.040*	*-.069*	*.076*	.113	*.036*	.221	.220	-.154	-.184	*-.080*	*.029*
8.	Environmental quality	5.905	1.396							—	.299	.179	-.173	*-.059*	*-.042*	*-.062*	*.040*	*.083*	.089	.153	.173	-.091	-.100	*-.049*	*.014*
9.	Security in daily life	6.535	0.842								—	.205	-.291	-.119	*-.048*	*-.059*	.181	.189	*.054*	.226	.199	-.246	-.233	*-.068*	*-.044*
10.	Physical condition	4.588	1.718									—	-.268	-.184	-.145	*-.077*	.116	.185	*.022*	.215	.151	-.259	-.112	*.050*	*-.007*
11.	Job strain	0.944	0.398										—	.377	.349	.297	-.408	-.177	-.245	-.230	-.141	.537	.090	-.108	*.063*
12.	Unreasonable work tasks	3.035	1.396											—	.529	.190	-.207	-.112	-.154	-.121	-.104	.324	*.081*	*-.044*	*-.008*
13.	Unnecessary work tasks	3.562	1.274												—	*.074*	-.286	-.176	-.239	-.112	*-.084*	.332	*.028*	*-.040*	*.029*
14.	Weekly working hours	38.210	10.954													—	-.157	*.021*	*.000*	*-.065*	*-.047*	.159	*-.040*	.158	*.056*
15.	Job autonomy	4.140	0.967														—	.249	.175	.223	.165	-.334	*-.049*	*-.004*	-.132
16.	Skill development	3.912	1.093															—	.313	.198	.199	-.150	*-.083*	*-.056*	-.094
17.	Performance feedback	2.554	1.270																—	.230	.179	-.183	*-.085*	*-.082*	*.007*
18.	Support in work life	5.405	1.617																	—	.762	-.267	-.292	*-.086*	-.196
19.	Support in personal life	5.821	1.448																		—	-.214	-.304	-.129	-.212
20.	Work-nonwork conflict	3.412	1.950																			—	.242	-.104	*-.035*
21.	Nonwork-work conflict	1.988	1.437																				—	*.011*	*-.053*
22.	Sex	0.319	0.467																					—	.189
23.	Age	44.951	10.539																						—

Notes. SA: sentimental accomplishment; FA: familial accomplishment. Correlations pertaining to sentimental accomplishment involved only 431 participants (about 16% of the Swiss participants were not married/sentimentally engaged); correlations pertaining to familial accomplishment involved only 365 participants (about 29% of the Swiss participants were childless). Nonsignificant correlations ($p > .05$) are italicized.
